# Thoracic SMARCA4-deficient undifferentiated tumor prone to misdiagnosis as lung adenocarcinoma: a case report and literature review

**DOI:** 10.3389/fonc.2026.1813580

**Published:** 2026-04-13

**Authors:** Xiuwen Yu, Ying Cai, Haiyan Wu, Hongsheng Liu, Bin Huang

**Affiliations:** 1 Department of Respiratory Medicine, The First People’s Hospital of Xiaoshan District, Hangzhou, Zhejiang, China; 2 Department of Pathology, The First People’s Hospital of Xiaoshan District, Hangzhou, Zhejiang, China

**Keywords:** lung adenocarcinoma, misdiagnosis, SMARCA4-deficient, thorax, undifferentiated tumor

## Abstract

Thoracic SMARCA4-deficient undifferentiated tumor (T-SMARCA4-DUT) is a rare neoplastic entity with a high risk of being misdiagnosed. We report the case of a 64-year-old male initially suspected of having mediastinal lymphoma by contrast-enhanced chest computed tomography (CECCT), and pathologically diagnosed with metastatic poorly differentiated lung adenocarcinoma via cervical lymph node biopsy. Initial immunohistochemical (IHC) results showed TTF-1 (clone SPT24) (+), Napsin A (-), p40 (-), and next-generation sequencing (NGS) detected KRAS amplification and TP53 missense mutation. Combined chemotherapy plus immunotherapy initially shrank the tumor, but bilateral axillary lymphadenopathy subsequently developed. Pathological analysis of axillary metastases and supplementary IHC of the original cervical biopsy revealed SMARCA4/Brg1 (-), SOX2 (+) and TTF-1 (clone 8G7G3/1) (-), leading to a revised diagnosis of lymph node-metastatic T-SMARCA4-DUT. This case identifies the non-specific clinical features of T-SMARCA4-DUT and an inadequate initial IHC panel as the core causes for misdiagnosis and confirms that a definitive diagnosis requires testing for SMARCA4/Brg1, SOX2, and the highly specific TTF-1 (clone 8G7G3/1).

## 1 Introduction

Thoracic SMARCA4-deficient undifferentiated tumor (T-SMARCA4-DUT) is a rare, highly malignant tumor newly categorized in the 2021 World Health Organization (WHO) Classification of Thoracic Tumors, previously designated as SMARCA4-deficient thoracic sarcoma (1, 2). It predominantly affects male patients with a long-term smoking history (1–3), who present with non-specific clinical symptoms including cough, chest tightness and unintentional weight loss. Imaging examinations usually reveal mediastinal or hilar masses, findings that overlap with those of lung cancer and lymphoma (4, 5). Tumor cells are undifferentiated round or epithelioid cells, often with rhabdoid differentiation and extensive tumor necrosis (1, 3).

IHC characterization of T-SMARCA4-DUT is marked by the loss or significant reduction of SMARCA4 expression, with occasional expression of CD34, SALL4 and SOX2 (6). A small number of cases show focal weak expression of lung cancer-associated markers (e.g., TTF-1, p40, CK) and rare PAX8 expression, further complicating the diagnostic process (3, 4, 7, 8). Currently, clinicians and pathologists have limited awareness of this rare tumor, and initial biopsy IHC panels typically focus on distinguishing lung adenocarcinoma from squamous cell carcinoma (e.g., TTF-1, Napsin A, p40) (9), often neglecting SMARCA4/Brg1 and SOX2 detection, which may cause delayed diagnosis or misdiagnosis.

T-SMARCA4-DUT exhibits highly malignant biological behavior, with most patients diagnosed at clinical stage IV and a median overall survival of only 4–7 months (2, 4, 8). Treatment regimens and prognostic outcomes differ significantly between T-SMARCA4-DUT and lung adenocarcinoma (especially those with driver gene mutations) or lymphoma, and misdiagnosis can directly lead to inappropriate treatment and delayed optimal therapy. We present a case of T-SMARCA4-DUT that was initially misdiagnosed as lung adenocarcinoma, analyze the causes of misdiagnosis, and discuss key differential diagnostic points via a literature review, aiming to provide practical references for reducing clinical and pathological misdiagnosis.

## 2 Case presentation

### 2.1 Clinical profile

A 64-year-old male with a 40-year smoking history (40 cigarettes per day) presented with dry cough and exertional chest tightness for over 8 months, accompanied by progressive weight loss in the prior 3 months. Two months prior to admission, he was diagnosed with chronic obstructive pulmonary disease and mediastinal lymphadenopathy, and was treated with budesonide/glycopyrronium/formoterol inhalation therapy. Four days before admission, right chest dull pain and aggravated cough prompted hospital admission for further evaluation.

Physical examination: Body temperature 36.9 °C, pulse 110 beats/min, respiratory rate 20 breaths/min, blood pressure 125/89 mmHg, room air oxygen saturation 95%. Two bean-sized hard, non-tender lymph nodes were found in the right supraclavicular fossa, and one bean-sized soft, non-tender lymph node in the left supraclavicular fossa. A barrel chest was observed with diminished bilateral breath sounds; no lower extremity edema was detected.

**Figure 1:**
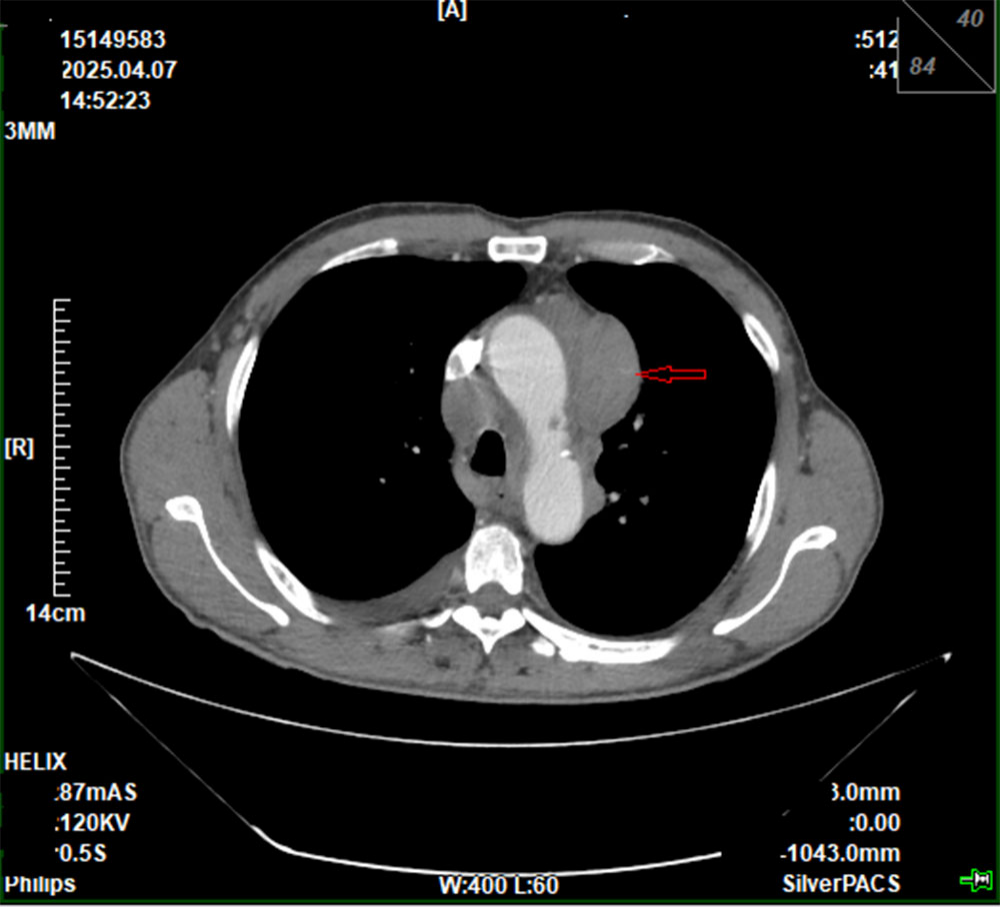
CECCT scan shows extensive enlargement and fusion of anterior mediastinal and bilateral hilar lymph nodes (red arrow, maximum diameter 5.2×3.1 cm).

CECCT (**Figure 1**) showed extensive enlargement and fusion of anterior mediastinal and bilateral hilar lymph nodes (maximum 5.2×3.1 cm), leading to an initial suspicion of mediastinal lymphoma, accompanied by pericardial effusion, bilateral pleural effusion, emphysema, and enlarged lymph nodes in the bilateral clavicular regions and axillae. Serum tumor markers: Carcinoembryonic antigen (CEA) 81.55 ng/mL (reference: 0.00-5.00 ng/mL), carbohydrate antigen 125 (CA125) 209.20 U/mL (reference: 0.00-35.00 U/mL), squamous cell carcinoma antigen (SCC) 0.25 ng/mL (reference: <1.50 ng/mL). The patient had no family history of malignant tumors.

Due to the high procedural risk of fiberoptic bronchoscopy after anesthetic evaluation, a cervical lymph node biopsy under local anesthesia was performed instead.

### 2.2 Disease timeline

1. August 2024: Dry cough and exertional chest tightness developed, without standardized treatment.
2. February 2025: Diagnosed with chronic obstructive pulmonary disease and mediastinal lymphadenopathy; anti-infective therapy was ineffective.
3. April 2025: Admitted for aggravated symptoms; CECCT suggested lymphoma. Cervical lymph node biopsy and NGS indicated stage IV metastatic poorly differentiated lung adenocarcinoma (KRAS amplification, TP53 mutation) with bone metastasis; chemotherapy + immunotherapy was initiated.
4. May 2025: Tumor shrinkage observed on non-CECCT 1 month after treatment initiation.
5. June 2025: Nearly complete tumor regression observed on non-CECCT 2 months after treatment initiation.
6. September 2025: 5 cycles of combined treatment completed; thrombocytopenia was managed symptomatically.
7. October-November 2025: Hoarseness and axillary masses developed; bilateral axillary lymphadenopathy was confirmed, and an axillary lymph node aspiration biopsy was performed. Pathological analysis of the biopsy and supplementary IHC revised the diagnosis to nodal metastatic T-SMARCA4-DUT; treatment adjusted to paclitaxel monotherapy.
8. December 2025 – February 2026: 3 cycles of paclitaxel monotherapy completed with symptomatic support and monitoring.
9. February 2026: Patient alive with tumor, continuing regular follow-up.

### 2.3 Pathology and genetic testing

Tissue specimens were fixed in 10% neutral buffered formalin at 25 °C for 24 h, embedded in paraffin, and cut into 3 µm thin sections. Hematoxylin and eosin (HE) staining was performed at 25 °C for 3 h (reagents from Shanghai Regal Biological Technology Development Co., Ltd. and Sinopharm Chemical Reagent Co., Ltd.). IHC staining was conducted via the EnVision system with antibodies from Beijing Zhongshan Jinqiao Biotechnology Co., Ltd. and Fuzhou Maixin Biotechnology Development Co., Ltd. NGS was performed at Hangzhou ADICON Clinical Laboratories Co., Ltd., Zhejiang, China.

**Figure 2:**
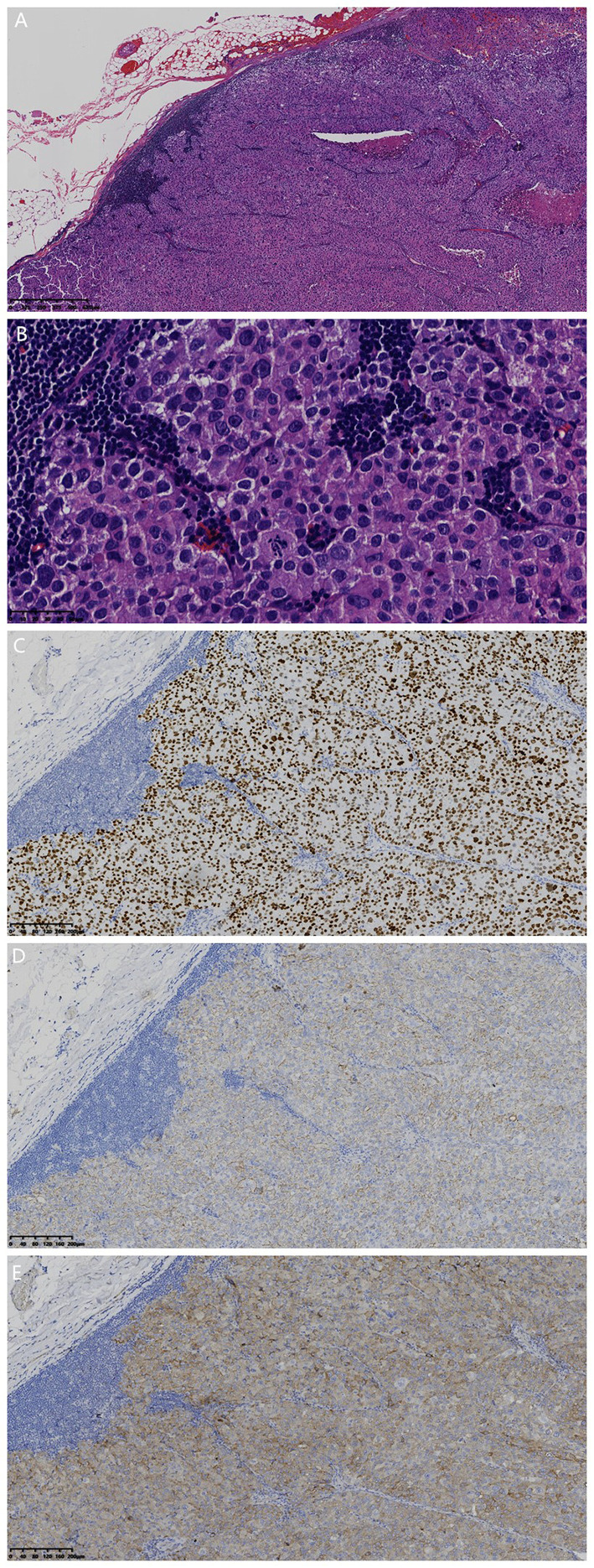
Histomorphological and IHC findings: **(A)** metastatic tumor in the right cervical lymph node (magnification, 32×; scale bar, 625 µm; hematoxylin and eosin staining); **(B)** tumor cells growing in solid nests with round to oval nuclei and frequent mitotic figures (magnification, 400×; scale bar, 50 µm; hematoxylin and eosin staining); **(C)** tumor cells with TTF-1 (SPT24) (+) (magnification, 100×; scale bar, 200 µm; EnVision immunohistochemistry kit); **(D)** tumor cells with CK (weak +) (magnification, 100×; scale bar, 200 µm; EnVision immunohistochemistry kit); **(E)** tumor cells with Synaptophysin (weak +) (magnification, 100×; scale bar, 200 µm; EnVision immunohistochemistry kit).

Cervical lymph node biopsy histological examination (**Figures 2A, B**) revealed metastatic tumor cells arranged in solid sheets, with round to oval nuclei of varying sizes, prominent nucleoli, frequent mitotic figures, and multifocal tumor necrosis. IHC results (**Figures 2C–E**): TTF-1 (SPT24) (+), CK (AE1/3) (weak +), CK7 (EP16) (focal +), Synaptophysin (UMAB112) (weak +), Napsin A (IP64) (-), p40 (ZR8) (-), Ki-67 (EP5) proliferation index 75%. The pathological diagnosis suggested lymph node metastatic poorly differentiated lung adenocarcinoma, and adenocarcinoma cells were identified in the pericardial effusion cytological examination.

NGS of 56 lung cancer-related genes detected KRAS amplification (mutation frequency >5%) and TP53 missense mutation (60.1%). Microsatellite instability (MSI) testing showed microsatellite stability (MSS). Whole-body bone scintigraphy revealed high osteoblastic activity in the fifth right anterior rib, suggesting bone metastasis.

### 2.4 Treatment and disease progression

The patient was diagnosed with lung adenocarcinoma (cT1N3M1c, stage IV) and received 5 cycles of chemotherapy with pemetrexed (0.8 g, day 1) + cisplatin (60 mg, days 1–2) combined with tislelizumab (200 mg, day 1), followed by tislelizumab maintenance therapy every 3 weeks as an outpatient. Thrombocytopenia occurred during treatment and was managed with recombinant human thrombopoietin.

**Figure 3:**
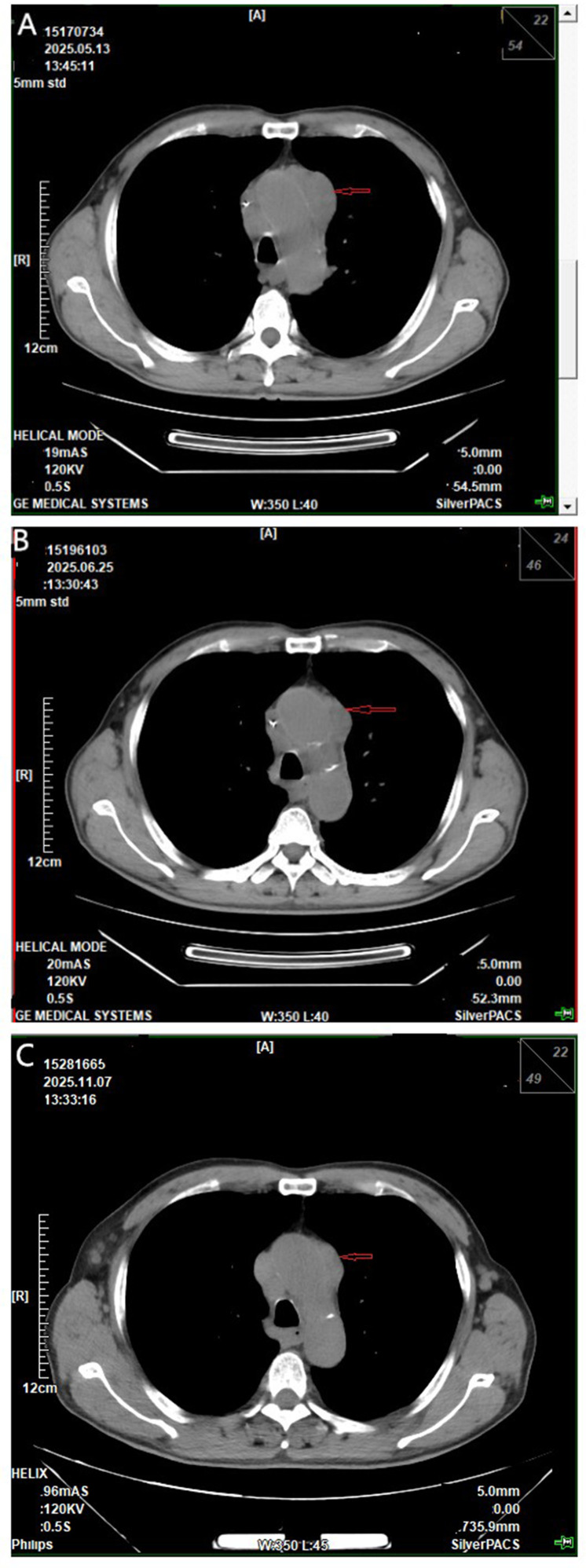
Non-CECCT scans: **(A)** the anterior mediastinal tumor (red arrow, maximum diameter: 3.9×1.4 cm) decreased in size after 1 month of treatment. **(B, C)** Nearly complete resolution of the anterior mediastinal tumor was achieved after two and seven months of treatment, respectively (red arrow).

Non-CECCT re-examination 1 month after treatment initiation showed the anterior mediastinal tumor shrank to 3.9×1.4 cm (**Figure 3A**). Non-CECCT re-examination at 2 and 7 months after treatment initiation demonstrated nearly complete resolution of the anterior mediastinal tumor (**Figures 3B, C**).

Six months after treatment initiation, the patient developed hoarseness and bilateral axillary masses. Otolaryngological consultation indicated right vocal cord paralysis, suspected to be secondary to recurrent laryngeal nerve compression. Ultrasonography confirmed bilateral axillary lymphadenopathy (right: 2.0×1.4 cm; left: 2.9×1.3 cm).

**Figure 4:**
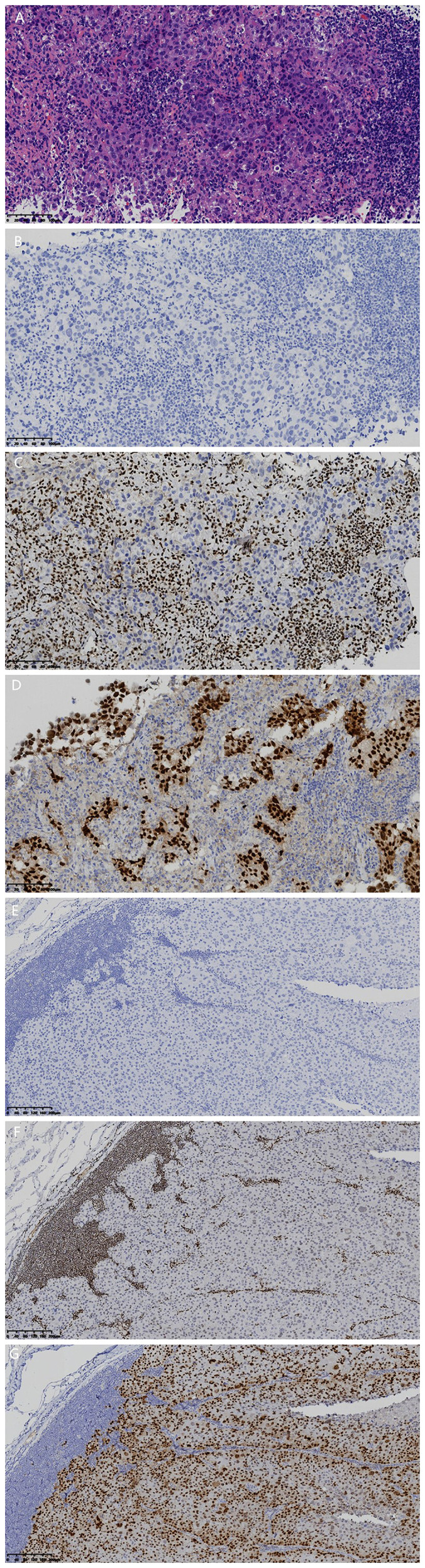
Histomorphological and IHC findings: **(A)** poorly differentiated metastatic carcinoma in the right axillary lymph node (magnification, 200×; scale bar, 100µm; hematoxylin and eosin staining); **(B)** tumor cells with TTF-1 (8G7G3/1) **(-)** (magnification, 200×; scale bar, 100 µm; EnVision immunohistochemistry kit); **(C)** tumor cells with SMARCA4/Brg1 (-), accompanied by internal control (lymphocytes +) (magnification, 200×; scale bar, 100 µm; EnVision immunohistochemistry kit); **(D)** tumor cells with SOX2 (+) (magnification, 200×; scale bar, 100 µm; EnVision immunohistochemistry kit); **(E)** supplementary IHC of tumor cells in cervical lymph node showing TTF-1 (8G7G3/1) (-) (magnification, 100×; scale bar, 200 µm; EnVision immunohistochemistry kit); **(F)** supplementary IHC of tumor cells in cervical lymph node showing SMARCA4/Brg1 (-), accompanied by internal control (lymphocytes +) (magnification, 100×; scale bar, 200 µm; EnVision immunohistochemistry kit); **(G)** supplementary IHC of tumor cells in cervical lymph node showing SOX2 (+) (magnification, 100×; scale bar, 200 µm; EnVision immunohistochemistry kit).

Right axillary lymph node aspiration biopsy pathological examination showed poorly differentiated metastatic carcinoma in lymphofibrous tissue (**Figure 4A**), with HE staining morphological features consistent with cervical metastatic carcinoma. Considering tumor heterogeneity, an extended IHC panel was tested, including TTF-1 (8G7G3/1), TTF-1 (SPT24), SMARCA4/Brg1 (E8V5B), SOX2 (EP103), SALL4 (EP103), and CD34 (QBEnd/10). IHC results (**Figures 4B–D**): TTF-1 (8G7G3/1) (-), focal TTF-1 (SPT24) (+), SMARCA4/Brg1 (-), SOX2 (+), SALL4 (-), CD34 (-), PD-L1 (TPS 50%).

Supplementary IHC staining of the initial cervical lymph node specimen confirmed TTF-1 (8G7G3/1) (-), SMARCA4/Brg1 (E8V5B) (-), and SOX2 (EP103) (+) (**Figures 4E–G**). The final pathological diagnosis was lymph node metastatic T-SMARCA4-DUT.

### 2.5 Subsequent treatment

A multidisciplinary discussion involving the departments of Respiratory Medicine, Medical Oncology, Radiotherapy, and Pathology concluded that the patient had disease progression due to suboptimal efficacy of the initial chemotherapy and immunotherapy regimen. Based on documented drug resistance, pharmacological properties, and the patient’s financial status, the treatment regimen was adjusted to single-agent paclitaxel chemotherapy. Paclitaxel was selected for its controllable hematological toxicity at the selected dose, which balances efficacy and safety, while novel targeted or investigational drugs were not feasible due to financial constraints. To date, the patient has completed 3 cycles of paclitaxel chemotherapy (300 mg, intravenous infusion on day 1), with concurrent nutritional support, symptomatic management, and routine monitoring (complete blood count, liver and renal function, CECCT).

### 2.6 Follow-up

The patient has been followed up for 10 months since the initial lung adenocarcinoma diagnosis and treatment initiation, with stable disease under current observation.

## 3 Discussion

T-SMARCA4-DUT is a rare, highly malignant tumor classified under “other epithelial tumors of the lung” in the 2021 WHO Classification (1). Definitive diagnosis often requires repeated biopsies, expanded IHC panels, and confirmation of SMARCA4 expression loss or gene mutation due to limited clinical and pathological data. Through this case report and literature review, we analyze the key clinical and pathological characteristics of this tumor, and provide references for clinical and pathological diagnosis.

### 3.1 Causes of misdiagnosis in this case

#### 3.1.1 Non-specific clinical and imaging features

The 64-year-old male patient with a long-term smoking history presented with cough, chest tightness, and weight loss; CECCT showed mediastinal lymph node enlargement with fusion, accompanied by pleural and pericardial effusion, mimicking mediastinal lymphoma. Significantly elevated serum CEA and CA125 levels were consistent with abnormalities seen in lung adenocarcinoma. These non-specific features provided spurious evidence for the initial misdiagnosis.

T-SMARCA4-DUT occurs in patients aged 27–90 years (median 48 years), with 90% occurring in heavy-smoking males (4, 8). Common symptoms include dyspnea, chest pain, weight loss, and superior vena cava syndrome, with imaging showing mediastinal or hilar tumors (4, 10). Its non-specific clinical and imaging manifestations easily lead to confusion with mediastinal lymphoma and common lung cancer subtypes.

#### 3.1.2 Inadequate initial IHC panel and misinterpretation of staining results

The initial diagnosis of lung adenocarcinoma was based on solid-growing round tumor cells with TTF-1 (SPT24) (+) and weak CK expression. In contrast, T-SMARCA4-DUT cells are characterized by undifferentiated high-grade morphology and a core IHC feature of SMARCA4/Brg1 loss or marked downregulation (1)—a finding in <10% of lung adenocarcinomas (11).

SMARCA4-deficient tumors lose typical epithelial features, often with weak/absent pan-cytokeratin expression (8). Here, weak CK (AE1/3) was misinterpreted as a feature of poorly differentiated lung adenocarcinoma, but it is a hallmark of SMARCA4 deficiency (a rare finding in classic poorly differentiated lung adenocarcinoma, which exhibits diffuse strong CK staining). The combination of weak CK (AE1/3) and Napsin A (-) should have raised suspicion for an uncommon thoracic malignancy, prompting SMARCA4/Brg1 and SOX2 testing—highlighting that IHC staining intensity/distribution, not just binary results, is critical for differential diagnosis. T-SMARCA4-DUT may express CD34, SOX2, and/or SALL4, while focal weak expression of TTF-1, p40, CK, and PAX8 is rare (3, 4, 7, 8).

Combined TTF-1 (preferentially clone 8G7G3/1) and p40 testing aids in diagnosing poorly differentiated NSCLC (12, 13). In this case, initial TTF-1 (SPT24) (+), subsequent TTF-1 (8G7G3/1) (-), supplementary SOX2 (+), and complete SMARCA4 loss confirmed the final diagnosis, though the specific TTF-1 clone linked to focal weak expression in reported T-SMARCA4-DUT cases remains unclear.

#### 3.1.3 Overlapping genomic mutations with non-small cell lung cancer

KRAS amplification (mutation frequency >5% here) differs from common KRAS activating point mutations (14): while point mutations are classic NSCLC drivers that activate the RAS-MAPK pathway, amplification causes gene dosage increase and protein overexpression, triggering pathway hyperactivation (14–16). KRAS amplification is rare in lung adenocarcinoma (1.1~3.1% incidence) (14, 17), and it coexisted with TP53 mutations—common in NSCLCs (49% (18)) and linked to poor prognosis.

Initially, these two genetic alterations—classic in smoking-related lung adenocarcinoma—were taken as confirmatory evidence for metastatic lung adenocarcinoma. The team focused solely on routine NSCLC IHC markers and ignored rare thoracic malignancies. Notably, KRAS (44%) and TP53 mutations also occur in T-SMARCA4-DUT (2, 8, 19), and their overlap with lung adenocarcinoma, coupled with the missing SMARCA4/Brg1 and SOX2 testing, further reinforced the misdiagnosis.

### 3.2 Treatment and prognosis

T-SMARCA4-DUT has an extremely poor prognosis, with a median overall survival of only 4–7 months. Surgery, radiotherapy, and conventional chemotherapy have limited efficacy, whereas chemotherapy combined with immunotherapy shows potential therapeutic value. PD-L1 overexpression [40%–60% of cases (3, 8)] is a common T-SMARCA4-DUT immunophenotype, which provides a biological basis for immune checkpoint inhibitor (ICI) application.

Consistent with this patient’s response, Song J et al. (3) reported a T-SMARCA4-DUT patient with PD-L1 TPS 30% who achieved 12-month radiological remission with carboplatin + etoposide + pembrolizumab; Rekhtman N et al. (8) noted that 3 of 11 T-SMARCA4-DUT patients receiving platinum-based chemotherapy combined with ICIs achieved sustained partial remission (>6 months). However, the survival-prolonging effect of these single-case/small-sample reports needs verification through large-sample cohort studies.

This patient achieved partial radiological remission (nearly complete anterior mediastinal tumor regression at 2 months) with first-line pemetrexed + cisplatin + tislelizumab, which is likely related to high PD-L1 expression (TPS 50%) in axillary metastases. Nevertheless, disease progression (axillary lymphadenopathy, recurrent laryngeal nerve compression) occurred at 6 months, consistent with T-SMARCA4-DUT’s highly malignant behavior—even with ICI combination therapy, most patients experience early progression due to complex drug resistance mechanisms (e.g., SMARCA4 deficiency-induced impaired antigen presentation, KRAS amplification-driven immune escape (11)). Notably, PD-L1 expression in the initial cervical metastases was not detected, and tumor heterogeneity may explain the inconsistent treatment responses between mediastinal and axillary lesions. After 6 months of initial combined treatment, the patient had disease progression; paclitaxel monotherapy achieved stable disease, and the patient has survived for an additional 3 months and is under ongoing follow-up.

### 3.3 Clinical and pathological recommendations

For thoracic masses with mediastinal lymphadenopathy and pleural effusion, in cases where pathological examination suggests poorly differentiated carcinoma and a definitive diagnosis cannot be made via routine IHC panels, an extended IHC panel including SMARCA4/Brg1, SOX2, CD34, and SALL4 should be applied.

The highly specific TTF-1 (clone 8G7G3/1) should be routinely used for TTF-1 detection to reduce misdiagnosis caused by inadequate marker panels. Genetic testing results should be comprehensively interpreted in combination with pathological morphology and immunophenotype. For unclear diagnoses, multidisciplinary team (MDT) discussions involving respiratory medicine, medical oncology, pathology, and radiology experts are recommended to achieve accurate diagnosis, formulate individualized treatment plans, and improve patient prognosis.

### 3.4 Limitations of the study

This single-case report has inherent limitations: first, the results and treatment experience cannot be generalized to all T-SMARCA4-DUT patients due to the disease’s rarity and significant clinical, morphological, and molecular heterogeneity; second, PD-L1 expression in the initial cervical metastases was not determined, and the lack of serial biopsies limits our understanding of tumor heterogeneity and drug resistance mechanisms; third, SMARCA4 gene sequencing was not performed to confirm the genomic basis of SMARCA4/Brg1 protein deficiency (e.g., frameshift mutations, nonsense mutations, copy number deletions), which would have provided more comprehensive molecular characteristics; fourth, the 10-month follow-up period is relatively short, and the long-term prognosis of paclitaxel monotherapy for T-SMARCA4-DUT remains unclear. Future multi-center, retrospective or prospective cohort studies with expanded sample sizes are needed to verify the proposed diagnostic process and treatment strategies, and explore the predictive value of molecular markers (e.g., PD-L1, SMARCA4 mutation type, KRAS status) for T-SMARCA4-DUT treatment responses.

## 4 Conclusion

This T-SMARCA4-DUT patient was initially misdiagnosed as lung adenocarcinoma due to non-specific clinical and imaging features, an inadequate initial IHC marker panel, and overlapping genomic mutations with NSCLC. Literature review indicates that T-SMARCA4-DUT misdiagnosis is closely related to the disease’s rarity and limited initial diagnostic testing panels. Clinicians and pathologists should enhance awareness of this rare tumor, optimize pathological testing panels (including timely SMARCA4/Brg1 detection and routine use of the highly specific TTF-1 (clone 8G7G3/1)), and promote MDT collaboration to achieve rapid and accurate diagnosis, formulate individualized reasonable treatment plans, and improve patient prognostic outcomes.

## Data Availability

The data analyzed in this study was obtained from Hangzhou ADICON Clinical Laboratories Co., Ltd., Zhejiang, China. The following restrictions apply: Data are not publicly available due to patient privacy and ethical constraints. Requests to access these datasets should be directed to Cuilian Sun, cuilian.sun@adicon.com.cn.
